# Protective efficacy of phosphodiesterase-1 inhibition against alpha-synuclein toxicity revealed by compound screening in LUHMES cells

**DOI:** 10.1038/s41598-017-11664-5

**Published:** 2017-09-13

**Authors:** Matthias Höllerhage, Claudia Moebius, Johannes Melms, Wei-Hua Chiu, Joachim N. Goebel, Tasnim Chakroun, Thomas Koeglsperger, Wolfgang H. Oertel, Thomas W. Rösler, Marc Bickle, Günter U. Höglinger

**Affiliations:** 1 Department of Translational Neurodegeneration, German Center for Neurodegenerative Diseases (DZNE), D-81377 Munich, Germany; 2 Department of Neurology, Technical University of Munich, D-81675 Munich, Germany; 3 HT-Technology Development Studio, Max Planck Institute of Molecular Cell Biology and Genetics, D-01307 Dresden, Germany; 4 Department of Neurology, University of Marburg, D-35043 Marburg, Germany; 5 Munich Cluster for Systems Neurology (SyNergy), D-81337 Munich, Germany; 6 Department of Neurology, Ludwig Maximilian University of Munich, D-81377 Munich, Germany; 7 Institute of Neurogenomics, Helmholtz Center Munich, D-85764 Neuherberg, Germany

## Abstract

α-synuclein-induced neurotoxicity is a core pathogenic event in neurodegenerative synucleinopathies such as Parkinson’s disease, dementia with Lewy bodies, or multiple system atrophy. There is currently no disease-modifying therapy available for these diseases. We screened 1,600 FDA-approved drugs for their efficacy to protect LUHMES cells from degeneration induced by wild-type α-synuclein and identified dipyridamole, a non-selective phosphodiesterase inhibitor, as top hit. Systematic analysis of other phosphodiesterase inhibitors identified a specific phosphodiesterase 1 inhibitor as most potent to rescue from α-synuclein toxicity. Protection was mediated by an increase of cGMP and associated with the reduction of a specific α-synuclein oligomeric species. RNA interference experiments confirmed PDE1A and to a smaller extent PDE1C as molecular targets accounting for the protective efficacy. PDE1 inhibition also rescued dopaminergic neurons from wild-type α-synuclein induced degeneration in the substantia nigra of mice. In conclusion, this work identifies inhibition of PDE1A in particular as promising target for neuroprotective treatment of synucleinopathies.

## Introduction

Parkinson’s disease (PD) is the most frequent neurodegenerative movement disorder. Its clinical core features are bradykinesia, rigidity, and tremor^1^. The major cause for these motor symptoms is the demise of dopaminergic neurons in the substantia nigra pars compacta. The current therapeutic approaches for PD are therefore mainly based on substitution of dopaminergic neurotransmission^2^. However, in more advanced disease stages, PD patients suffer from a broad spectrum of non-motor symptoms, including psychosis and cognitive decline, related to neurodegeneration in extended brain areas, including the amygdala and the cerebral cortex^3^. The histopathological hallmarks of PD are intracellular proteinaceous inclusions termed Lewy bodies, which consist mainly of aggregated α-synuclein (α-Syn)^4^. α-Syn is a 140 amino acid-long presynaptic protein of unknown physiological function^5^. Duplication, triplication, or point mutations of the *SNCA* gene encoding α-Syn are causative for dominantly inherited forms of PD^6–10^. Moreover, genome-wide association studies found variants of *SNCA* as major risk factors for sporadic PD^11^. Other synucleinopathies are dementia with Lewy bodies, characterized by early neocortical neuronal α-Syn pathology, and multiple system atrophy, characterized by glial cytoplasmic α-Syn inclusions. The α-Syn species that confer to toxicity are still under debate^12^. Some studies show that oligomers are toxic^13^ while others report that fibrillary α-Syn is toxic^14^. Moreover, it was shown that different α-Syn species found in different synucleinopathies, so-called strains, have different effects when administered to cultured cells or mice^15^. Furthermore, it was previously shown that mouse α-Syn interacts with human α-Syn and affects aggregation^16^. This demonstrates that the exact nature of the pathogenic α-Syn species and the mechanisms leading to cell death are not yet fully understood. However, different strategies targeting α-Syn are in the preclinical and clinical development^17^. *In vitro* data suggest that a stimulation of α-Syn degradation, e.g. by activation of autophagy, might be a promising approach to reduce the α-Syn burden^18^. Also, stimulation of glucocerebrosidase in α-Syn overexpressing cells with ambroxol reduced α-Syn levels^19^. Another strategy is the inhibition of α-Syn aggregation. Epigallocatechin gallate extracted from green tea, which has an inhibitory effect on α-Syn aggregation, is currently in clinical testing in patients with multiple system atrophy^20^. Moreover, strategies to reduce α-Syn propagation are under development, including passive^21^ and active immunisation^22^.

All synucleinopathies are relentlessly progressive. Despite the approaches described above, there is currently no known therapy with proven efficacy to slow or halt their progression, since all clinical trials with potentially neuroprotective interventions failed so far to show any disease modifying effects in synucleinopathies (e.g. refs 23 and 24).

Therefore, the development of new disease-modifying therapeutic strategies is of utmost importance.

To identify novel therapies against α-Syn-induced neurodegeneration, we have developed a model in which moderate overexpression of wild-type α-Syn with adenoviral vectors in postmitotic dopaminergic Lund human mesencephalic (LUHMES) neurons *in vitro* leads to ~50% cell death within six days^18^. In the present study, we miniaturized and automatized this model to perform a screening of 1,600 FDA-approved drugs.

## Results

### Screening of FDA-approved drugs for neuroprotective efficacy against α-Syn

**Figure 1:**
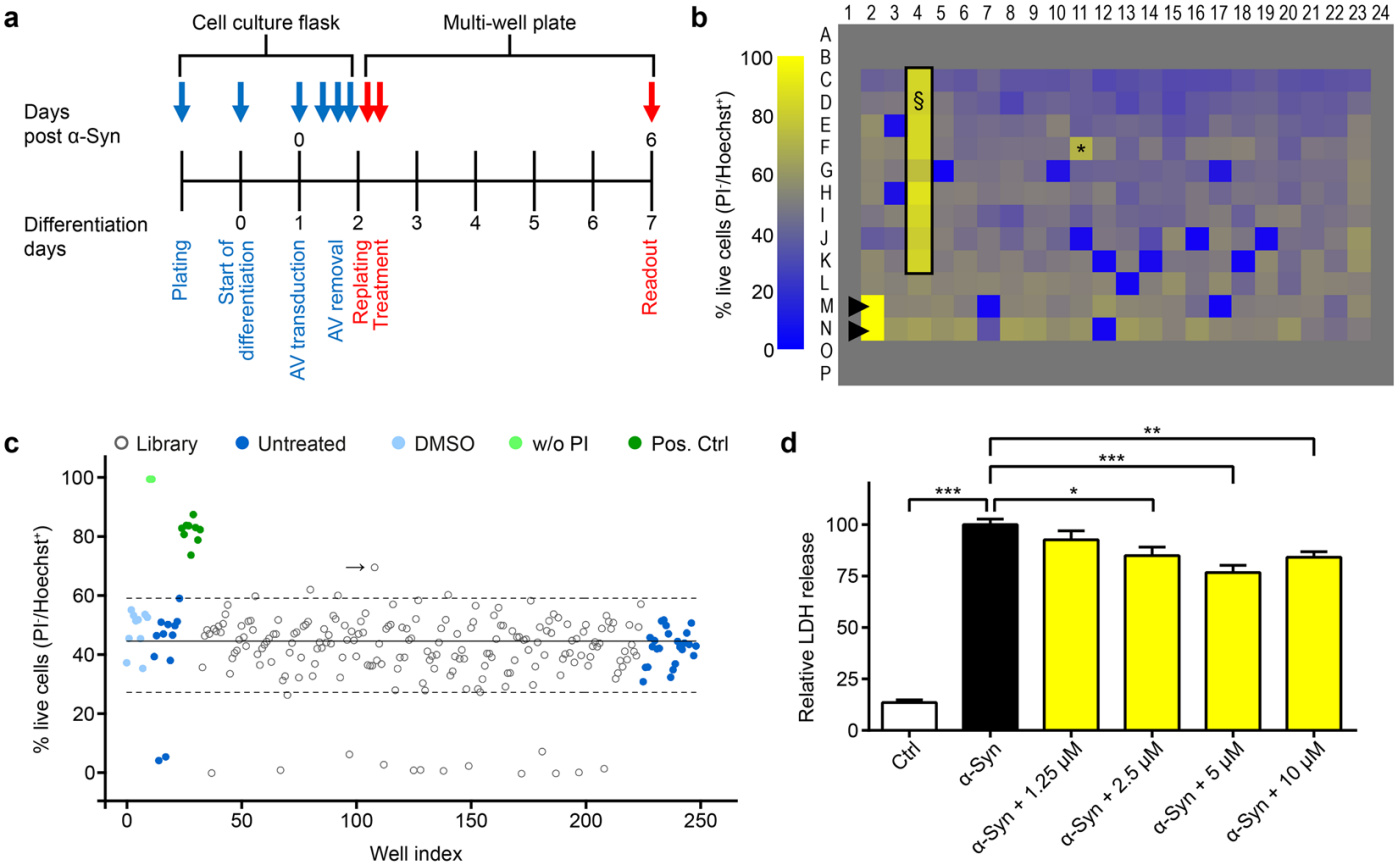
High-throughput screening of 1,600 FDA-approved drugs for modulators of α-Syn toxicity in postmitotic human dopaminergic mesencephalic LUHMES neurons. (**a**) Experimental design. Cells were first transduced in culture flasks (blue arrows), then transferred for screening to multi-well plates (red arrows). AV = adenoviral vectors encoding wild type α-Syn. (**b**) Representative heat map showing cell survival of α-Syn transduced neurons in colour codes ranging from yellow (100% survival) to blue (0% survival). The outermost (grey) wells contained no cells. Hoechst 33342 was used to label all cells, propidium iodide (PI) to label dead cells only. Survival rates were quantified as percentage of PI^−^ cells of all Hoechst^+^ cells. PI was omitted in the two wells marked with ▶(100% survival controls). The black frame (§) denotes wells treated with a previously identified protective compound (positive control). *Denotes a hit compound providing neuroprotection. (**c**) Representative scatterplot showing cell survival of **b** quantitatively. Continuous line: mean survival of α-Syn transduced neurons. Upper dashed line: Z-score of +2.5 (threshold for positive hits). Lower dashed line: Z-score of −3 (threshold for negative hits). Bright blue dots: wells treated with solvent (DMSO) only. Bright green dots: wells without PI. Dark blue dots: wells without treatment. Dark green dots: wells treated with a protective compound (positive control). Blank dots: wells treated with compounds from the library. →: hit compound (corresponding to * in **b**). (**d**) Concentration-dependent protective efficacy of the top hit dipyridamole against α-Syn-induced toxicity, measured by LDH release. Blank column: untransduced cells (Ctrl). Black column: α-Syn overexpressing cells without treatment. Yellow columns: α-Syn overexpressing cells treated with 1.25 µM to 10 µM of dipyridamole. *P < 0.05, **P < 0.01, ***P < 0.001, one-way ANOVA with Tukey’s HSD post-hoc test. *N* > 12, *F* = 97.74, degrees of freedom = 96. Data are mean ± SEM.

The recently reported α-Syn model^18^ was modified for high-throughput screening. In brief, LUHMES cells were differentiated into a postmitotic dopaminergic phenotype and transduced with adenoviral vectors to overexpress wild-type α-Syn (Fig. 1a). Cell death, quantified by automated high-throughput microscopy, was approximately 50% after 6 days of α-Syn overexpression.

We screened 1,600 FDA-approved drugs for their protective efficacy at concentrations of 3 µM and 10 µM, each in triplicates. In total six screening runs (three at each concentration) were performed for each compound. A representative overview of one of the screening plates is presented in Fig. 1b. A typical scatterplot illustrating quantitatively the cell survival rate in the individual wells of a plate is provided in Fig. 1c. Compounds were considered as positive (protective) hit if their Z-score was higher than +2.5. A negative (toxic) hit was defined by a Z-score below −3. The false-positive rate (i.e. percentage of wells treated with DMSO only scored as positive hit) was 0.8%. The false-negative rate (i.e. percentage wells treated with DMSO only scored as negative hits) was 0.4%.

In total, this primary screening identified 53 compounds that were protective in at least one of the six runs (40 at 3 µM, 22 at 10 µM, and 9 at both concentrations).

### Dipyridamole protects from α-Syn-induced toxicity

The most convincing hit was dipyridamole, since it was the only compound to be protective in all six runs. The compound is an unspecific inhibitor of phosphodiesterases (PDEs). The protective effect of dipyridamole was validated using an independent measure of cell death, i.e. the quantification of lactate dehydrogenase (LDH) release from α-Syn overexpressing LUHMES neurons into the cell culture medium. At 5 µM, dipyridamole reduced the LDH levels to 77.0 ± 5.1% compared to solvent (DMSO) treatment (*P* < 0.001, Fig. 1d). Thus, dipyridamole was the most promising candidate from the primary screening.

### LUHMES neurons express several PDEs

**Figure 2:**
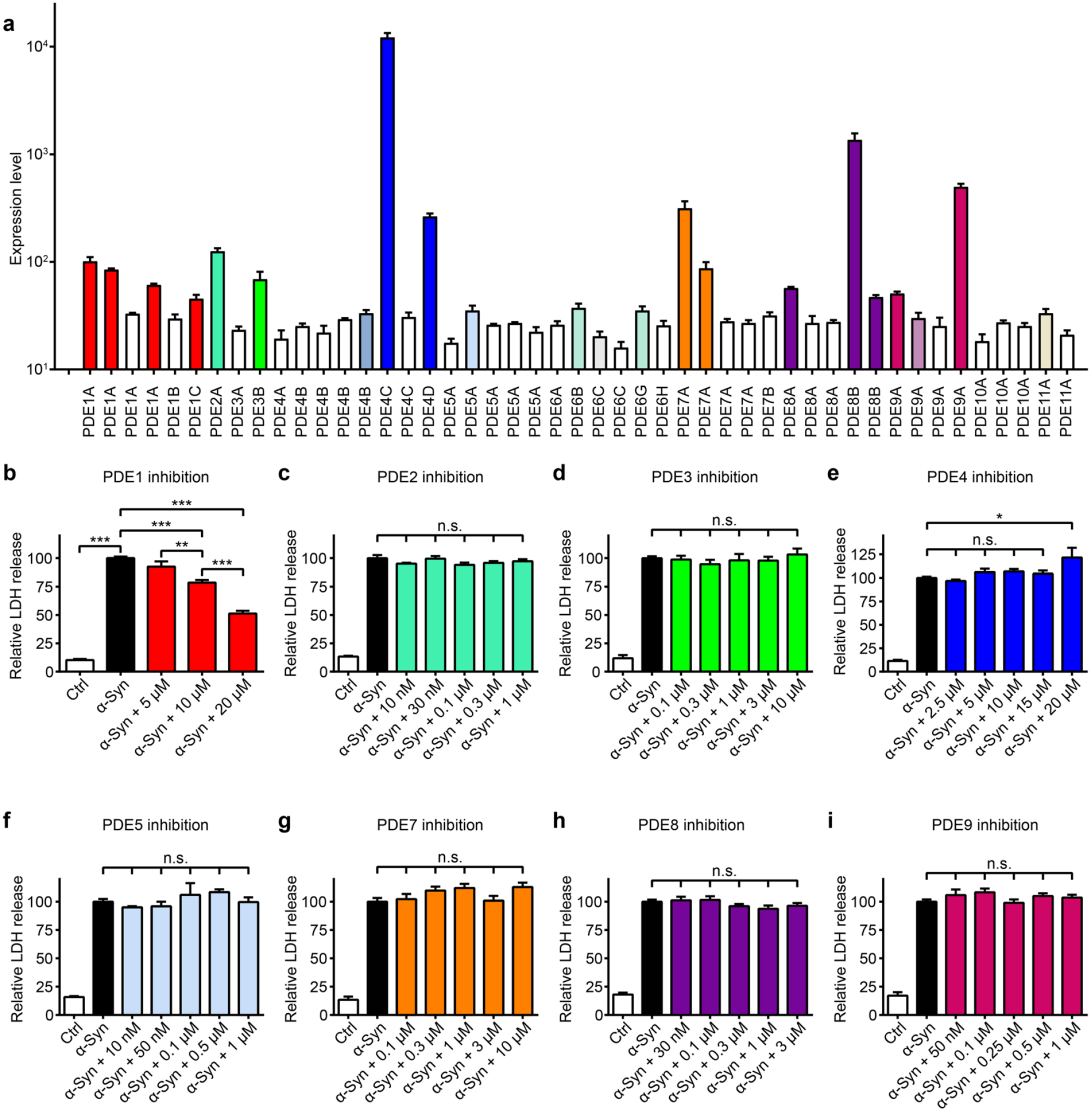
Phosphodiesterase (PDE) 1 inhibition protects against α-Syn toxicity. (**a**) Expression of the known PDE isoforms in LUHMES neurons four days after transduction with α-Syn. Bars in bright colours indicate PDEs with solid expression (detection p-value < 0.05 in all three samples). Blank bars indicate PDEs below the detection threshold (p-detection value < 0.05 in none of the samples). Pale bars indicate inconstant expression levels (detection p-value < 0.05 in one or two of three samples). Each bar represents a probe sequence on the Illumina chip that was assigned to a PDE. (**b**–**i**) Efficacy of different PDE inhibitors to prevent α-Syn-induced cell death was measured by LDH release. Data indicate untransduced control neurons (Ctrl, white bars), neurons overexpressing α-Syn treated with solvent only (black bars), or with different PDE inhibitors in their pharmacologically active range of concentrations (coloured bars): PDE1: vinpocetine, PDE2: Bay60-7550, PDE3: milrinone, PDE4: rolipram, PDE5: sildenafil, PDE7: BRL50481, PDE8: PF-4957325-00, PDE9: Bay73-6691. Only vinpocetine protected from α-Syn-induced toxicity. Data are normalized to LDH release in α-Syn-transduced cells with solvent treatment. *P < 0.05, **P < 0.01, ***P < 0.001, n.s. not significant, one-way ANOVA with Tukey’s HSD post-hoc test. N-values, F-values and degrees of freedom (DF): (**b**) *N* ≥ 24, *F* = 215.3 DF = 127, (**c**) *N* = 3, *F* = 300.7, DF = 14, (**d**) *N* = 9, *F* = 71.52, DF = 56, (**e**) *N* ≥ 9, *F* = 62.27, DF = 74, (**f**) *N* = 3, *F* = 47.23, DF = 14, (**g**) *N* = 9, *F* = 90.41, DF = 56, (**h**) *N* ≥ 9, *F* = 224.3, DF = 128, (**i**) *N* ≥ 9, *F* = 124.2, DF = 71. Data are mean ± SEM.

Since dipyridamole does not pass the blood-brain barrier, it does not qualify as compound for treatment of PD patients. We therefore studied its mechanism of action to identify the molecular target accounting for its neuroprotective efficacy. Dipyridamole is an unspecific inhibitor of PDEs^25, 26^, a superfamily of enzymes catalysing the degradation of cyclic adenosine monophosphate (cAMP) and/or cyclic guanosine monophosphate (cGMP). Hence, we investigated whether inhibition of specific PDE isoforms would confer protection against α-Syn-induced neurodegeneration. First, we analysed the expression of the PDE isoforms in our PD cell model using Illumina HumanHT-12_V3 bead chips (Illumina Inc., San Diego, CA, USA). A detection p-value below 0.05 as processed by Genome Studio Software (Illumina Inc.) was considered as statistically significant expression. Four days after adenoviral α-Syn transduction, cells strongly expressed PDE1A, PDE1C, PDE2A, PDE3B, PDE4C, PDE4D, PDE7A, PDE8A, PDE8B, and PDE9A (detection p-value < 0.05 in all three samples), and inconsistently expressed PDE4B, PDE5A, PDE6B, PDE6G, PDE11A (detection p-value < 0.05 in one or two of three samples, Fig. 2a). Thus, we concluded that these PDEs were the most promising targets for further investigation.

### The PDE1 inhibitor vinpocetine rescues LUHMES neurons from α-Syn-induced toxicity

We then examined the efficacy of specific pharmacological inhibitors^27^ of the expressed PDEs to reduce α-Syn-induced toxicity, quantified by LDH release. The PDE1 inhibitor vinpocetine led to a significant protection in a concentration-dependent manner against α-Syn-induced toxicity (Fig. 2b). Inhibitors of PDE2 (Bay60–7550), PDE3 (milrinone), PDE4 (rolipram), PDE5 (sildenafil), PDE7 (BRL 50481), PDE8 (PF-4957325-00), and PDE9 (Bay73-6691) did not protect (Fig. 2c–i). In absence of specific inhibitors, PDE6 and PDE11 were not tested. These findings suggest that particularly the isoenzyme PDE1 might be a relevant therapeutic target and that the blood-brain barrier permeable inhibitor vinpocetine was a promising candidate for further exploration.

### Validation of the protective efficacy of vinpocetine *in vitro*

**Figure 3:**
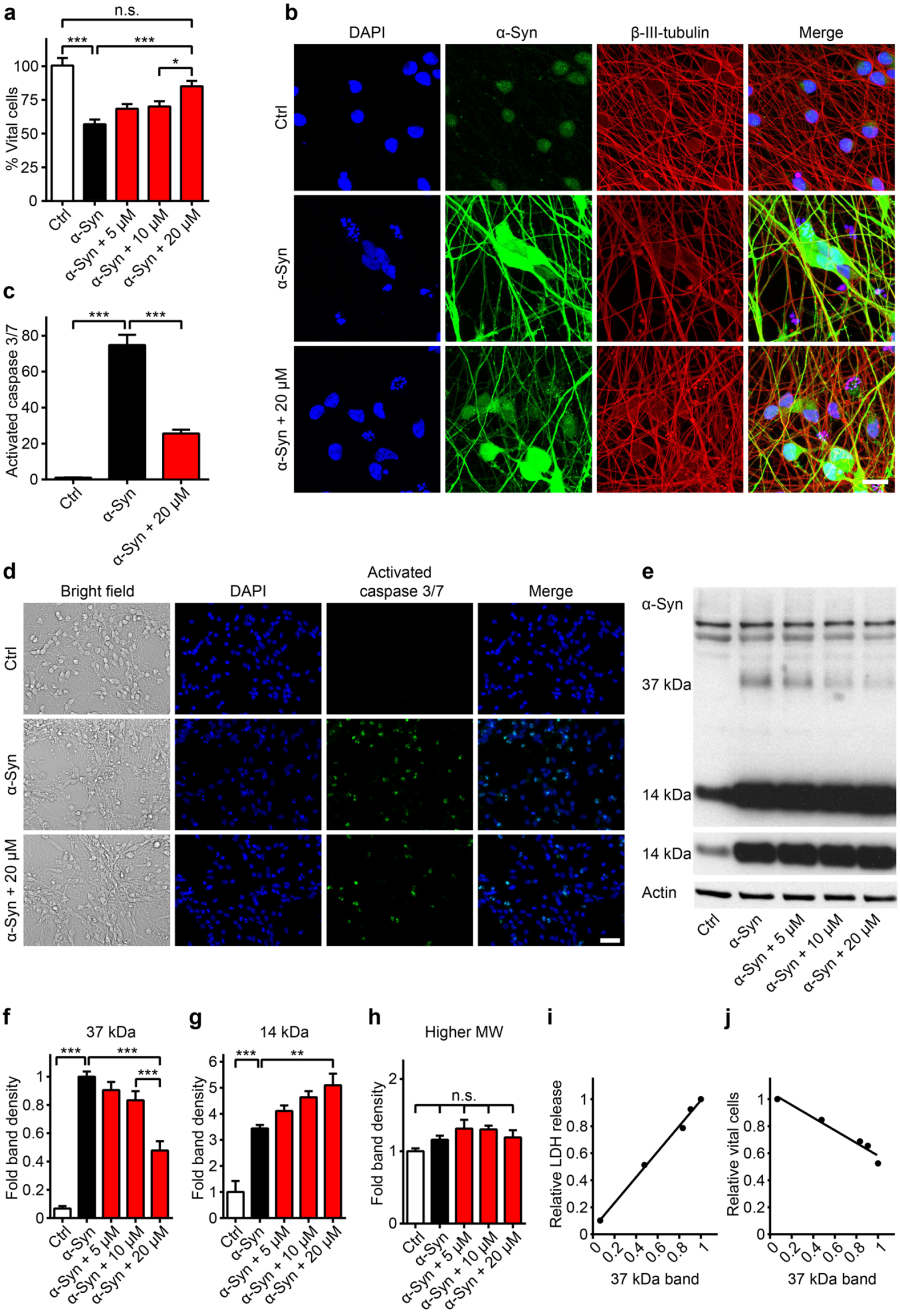
The PDE1 inhibitor vinpocetine reduces cell death and a 37 kDa α-Syn species. (**a**) Counts of vital, i.e. non-clumped, non-fragmented 4′,6-diamidino-2-phenylindole (DAPI)-stained nuclei in untransduced control neurons (Ctrl, white bar), α-Syn transduced neurons (black bar) treated with solvent only, and α-Syn transduced neurons treated with different concentrations of vinpocetine (red bars) confirmed the compound’s protective efficacy. (**b**) Confocal microscopy images of untransduced control neurons, α-Syn-transduced neurons treated with solvent, and α-Syn-transduced neurons treated with vinpocetine six days after transduction, stained with DAPI (blue), an α-Syn-antibody (green), and a β-III-tubulin-antibody to demonstrate the axonal network (red). Scale bar: 20 µm. (**c**) Quantification of the activated caspases signal after CellEvent^TM^ staining in untransduced control neurons (left bar), α-Syn-transduced neurons treated with solvent (black bar), and α-Syn-transduced neurons treated with vinpocetine (red bar) showing activation of caspases 3 and 7 in α-Syn overexpressing neurons, which was reduced by treatment with 20 µM vinpocetine. (**d**) Representative images of the CellEvent^TM^ staining. Scale bar 50 µm. (**e**) Representative Western blot with an α-Syn antibody (C20, Santa Cruz) in lysates of control neurons and α-Syn-transduced neurons treated with solvent or with vinpocetine at different concentrations. The upper panel shows a higher exposed image, the lower panel shows a lower exposed image. (**f**,**g**) Quantification of specific α-Syn Western blot bands showed that vinpocetine reduced a 37 kDa α-Syn band (**f**) increased the monomer α-Syn band (**g**), but did not change other α-Syn bands (**h**). The density of the 37 kDa band positively correlated with the LDH release (*r* ^2^ = 0.99, *P* < 0.001, **i**) and negatively correlated with the number of vital cells (*r* ^2^ = 0.96, *P* < 0.01, **j**). *P < 0.05, **P < 0.01, ***< 0.001, n.s. not significant, one-way ANOVA with Tukey’s HSD post-hoc test. N-values, F-values and degrees of freedom (DF): (**a**) *N* ≥ 56, *F* = 15.71, DF = 351, (**c**) *N* = 9, *F* = 115.9, DF = 24, (**f**) *N* ≥ 6, *F* = 45.91, DF = 34, (**g**) *N* ≥ 6, *F* = 23.59, DF = 34, (**h**) *N* ≥ 6, *F* = 2.46, DF = 34. Data are mean ± SEM. Full length Western blots are shown in Supplementary Fig. S5.

We aimed to confirm the efficacy of vinpocetine to protect neurons against α-Syn overexpression with independent methods. First, we determined the number of vital neuronal nuclei (i.e. non-clumped, non-fragmented). Overexpression of α-Syn reduced their number to 57.0 ± 3.4% of untransduced controls (*P* < 0.001), while co-treatment with 20 µM vinpocetine increased their number to 85.1 ± 4.1% (*P* < 0.001, Fig. 3a,b), confirming the LDH-data. Secondly, we used the CellEvent^TM^ dye to visualize activation of caspases 3 and 7 as marker for apoptosis. α-Syn overexpression strongly increased the CellEvent^TM^ signal 74.8 ± 5.7-fold vs. untransduced cells (*P* < 0.001), while co-treatment with 20 µM vinpocetine reduced the signal to 25.6 ± 2.1-fold (*P* < 0.001, Fig. 3c,d). These findings confirm that vinpocetine indeed prevents α-Syn-induced neuronal cell death *in vitro*. In untransduced or GFP transduced control cells, vinpocetine had no influence on cell viability (Supplementary Fig. S1a).

### Vinpocetine reduces the density of a 37 kDa α-Syn band

Previously, we described a 37 kDa α-Syn Western blot band in neurons occurring after α-Syn overexpression, the intensity of which correlated with neurodegeneration^18^. Thus, we studied if vinpocetine would influence the α-Syn Western blot pattern. As described^18^, the 37 kDa α-Syn band occurred upon α-Syn overexpression (Fig. 3e). Vinpocetine reduced this band in a concentration-dependent manner (by 52.2 ± 6.6% at 20 µM, *P* < 0.001, Fig. 3e,f). α-Syn overexpression expectedly increased the monomeric 14 kDa α-Syn band (3.4 ± 0.1-fold vs. untreated controls, *P* < 0.001), and vinpocetine treatment further increased this band (5.1 ± 0.4-fold, *P* < 0.01, Fig. 3g). Other α-Syn bands remained unchanged by vinpocetine (Fig. 3h). However, vinpocetine did not significantly alter levels of endogenous α-Syn in untransduced cells (Supplementary Fig. S1c,d). The presence of the 37 kDa band in α-Syn overexpressing cells and the reduction with vinpocetine was confirmed using a second anti-α-Syn antibody (Supplementary Fig. S2a). The density of the 37 kDa band, but not the 14 kDa band, correlated positively with LDH release (*r* ^2^ = 0.99, *P* < 0.001, Fig. 3i) and negatively with the number of vital cells (*r* ^2^ = 0.96, *P* < 0.01, Fig. 3j). These data might suggest an implication of the 37 kDa α-Syn band in α-Syn-induced toxicity.

### Mechanisms of action relevant for vinpocetine’s efficacy

**Figure 4:**
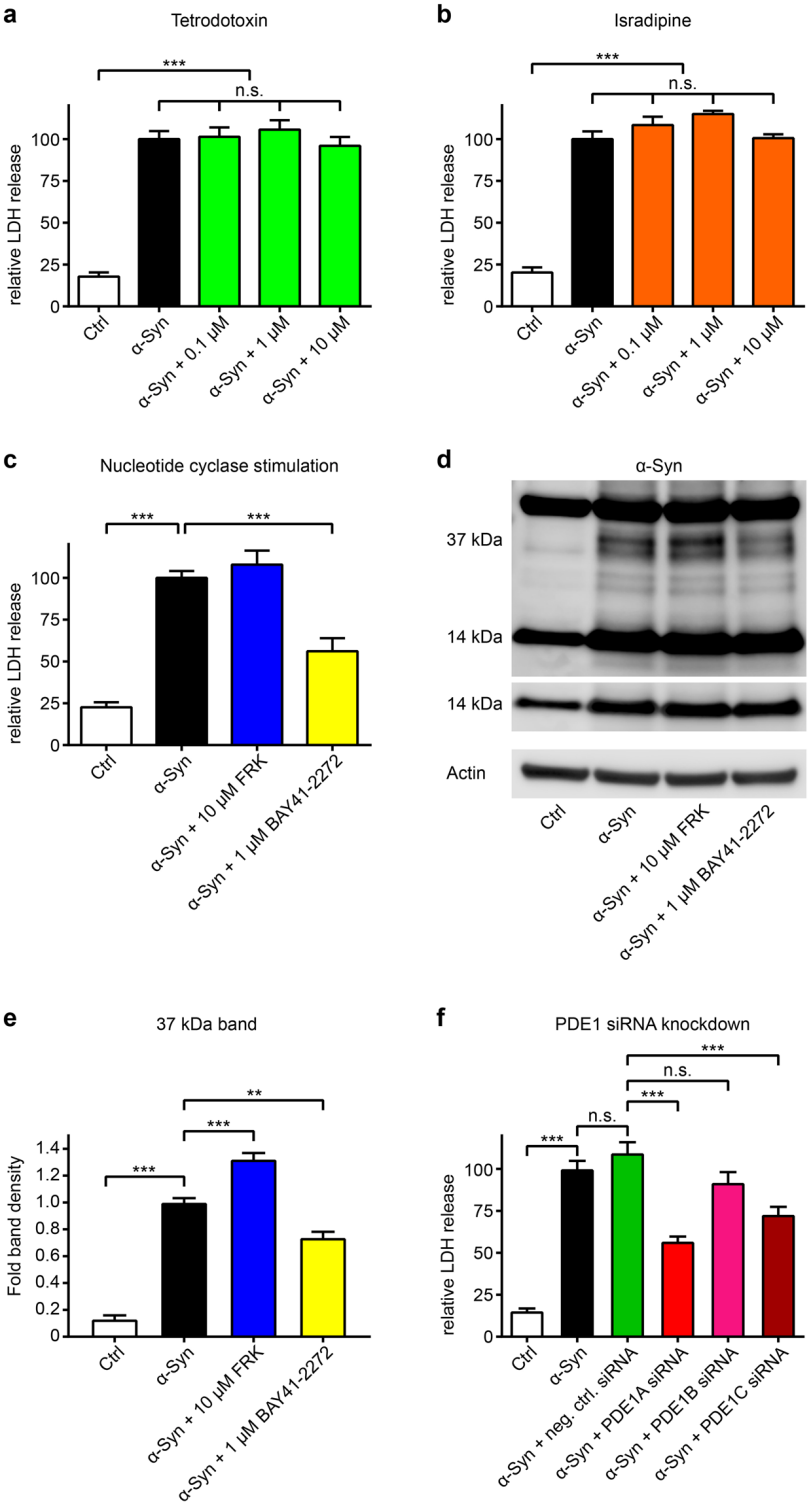
Mechanisms of action relevant for vinpocetine’s efficacy. (**a**–**d**) LDH release was used to measure degeneration in untransduced control neurons (Ctrl, white bars), α-Syn-transduced neurons treated with solvent (black bars), and α-Syn-transduced neurons with different interventions (coloured bars). The sodium channel blocker tetrodotoxin (**a**, green bars) and the L-type calcium channel blocker isradipine (**b**, orange bars) in different concentrations did not protect against α-Syn-incuded toxicity. The adenylate cyclase stimulator forskolin (**c**, FRK, blue bar) did also not protect, but the guanylate cyclase stimulator BAY41-2272 did (**c**, yellow bar). (**d**) Western blot with an α-Syn antibody (C20, Santa Cruz) of untransduced control neurons (Ctrl), α-Syn-transduced neurons treated with solvent (second lane), and α-Syn-transduced neurons with forskolin (FRK) or BAY41-2272 showed that BAY41-2272 treatment also led to a reduction of a 37 kDa α-Syn band, while forskolin led to an increase of this band. (**e**) Quantification of the 37 kDa band. (**f**) A negative control siRNA (green bar) and siRNA against PDE1B (pink bar) did not protect, but siRNA against PDE1A (bright red bar) was strongly protective, and siRNA against PDE1C (dark red bar) was moderately protective. *P < 0.05, **P < 0.01, ***P < 0.001, n.s. not significant, one-way ANOVA with Tukey’s HSD post-hoc test. N-values, F-values and degrees of freedom (DF): (**a**) *N* = 7, *F* = 116.1, DF = 30, (**b**) *N* = 6, *F* = 56.97, DF = 25, (**c**) *N* = 9, *F* = 40.5, DF = 32, (**d**), *N* = 4, *F* = 168.4, DF = 12, (**f**) *N* ≥ 9, *F* = 69.79, DF = 92. Data are mean ± SEM. Full length Western blots are shown in Supplementary Fig. S6.

Besides being a PDE1 inhibitor, vinpocetine also inhibits sodium channels^28^ and L-type calcium channels^29^. Thus, we studied if these mechanisms of action would be relevant for vinpocetine’s protective efficacy. Therefore, LUHMES cells overexpressing α-Syn were treated with the sodium channel blocker tetrodotoxin and the L-type calcium channel blocker isradipine. Both compounds did not protect against α-Syn-induced toxicity, demonstrating that cation channel blockage was not sufficient to explain vinpocetine’s protective efficacy (Fig. 4a,b).

Furthermore, PDE1 can degrade cAMP as well as cGMP. Therefore, we treated α-Syn-overexpressing LUHMES cells with the adenylate cyclase stimulator forskolin to increase cAMP^30^ or the guanylate cyclase stimulator BAY41-2272 to increase cGMP^31^. Forskolin did not influence α-Syn-induced toxicity, but BAY41-2272 reduced the relative LDH release to 56.2 ± 7.8% of untreated α-Syn overexpressing cells (*P* < 0.001, Fig. 4c). Moreover, in a similar way as vinpocetine, BAY41-2272 treatment also led to the reduction of a 37 kDa α-Syn (Fig. 4d,e), while forskolin led to an increase of this band. This was confirmed with a second anti-α-Syn antibody (Supplementary Fig. S2b). This suggested that the protective effect observed after PDE1 inhibition depended on cGMP rather than cAMP levels.

To exclude that the effect observed after treatment with vinpocetine, or BAY41-2272 was mediated by an influence on the adenoviral transduction, we performed Western blots in GFP-overexpressing cells. We did not see any differences of GFP levels after treatment with vinpocetine suggesting that the compounds did not affect the adenoviral transduction or expression of the protein itself (Supplementary Fig. S1).

The PDE1 subfamily comprises three isoforms: PDE1A, PDE1B, and PDE1C. qPCR confirmed that α-Syn-transduced LUHMES cells express PDE1A and PDE1C, but not PDE1B (Supplementary Fig. S3a). Since specific pharmacological inhibitors of these are not available, we knocked down PDE1A, PDE1B, and PDE1C with siRNAs. Silencing efficacies of the siRNAs against PDE1A and PDE1C were verified by qPCR (Supplementary Fig. S3b). Knock down of PDE1A, and to a lesser degree PDE1C, protected against α-Syn-induced toxicity (Fig. 4f). Since PDE1B was not expressed in LUHMES cells, the PDE1B siRNA was not protective. These data demonstrate that PDE1A and PDE1C isoforms are the relevant therapeutic targets of vinpocetine.

### Vinpocetine protects from α-Syn induced nigral neurodegeneration *in vivo*

**Figure 5:**
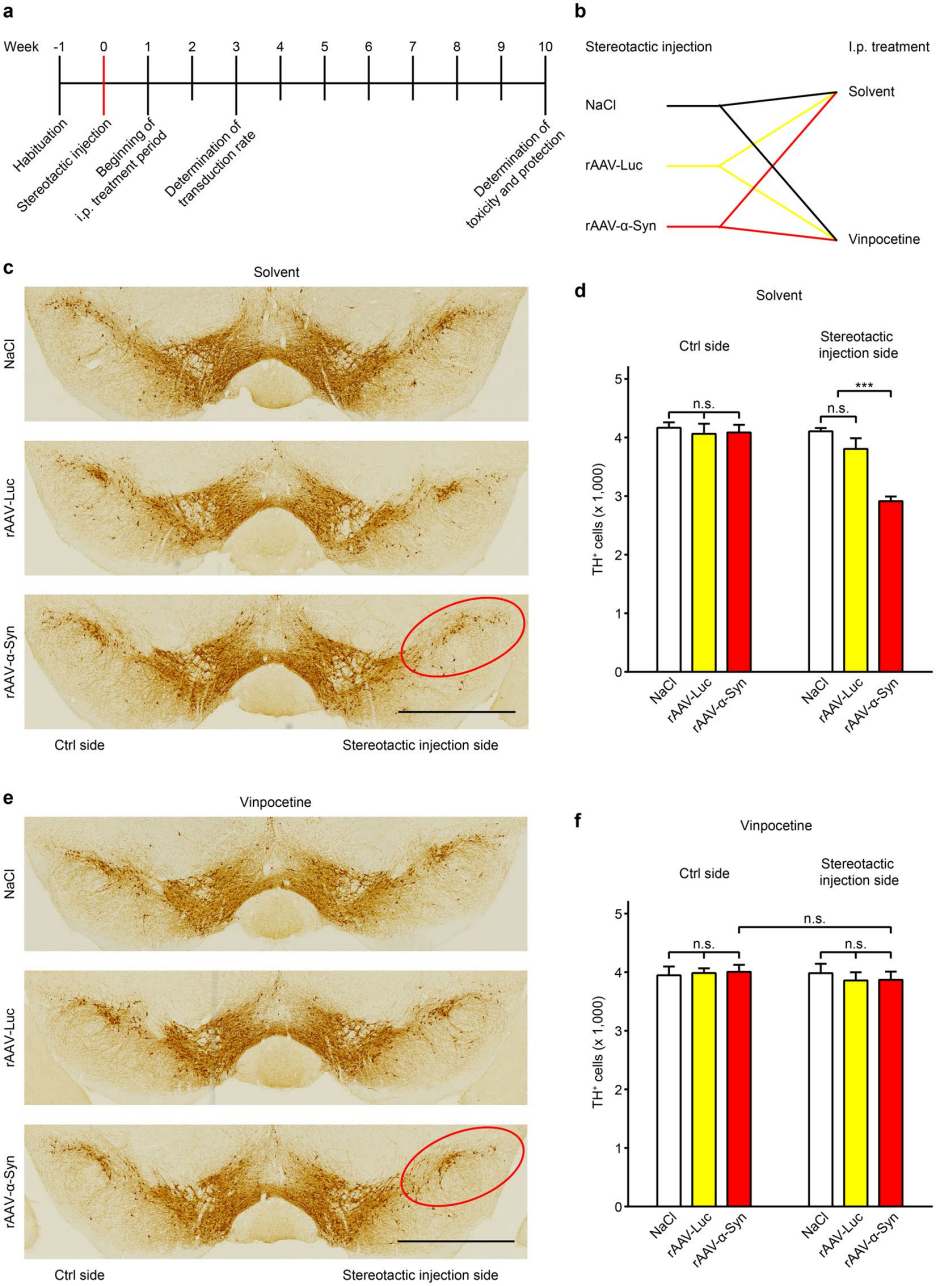
Vinpocetine protects against α-Syn-induced neurotoxicity in mice *in vivo*. (**a**) Experimental timeline and (**b**) experimental groups. Mice were injected stereotactically with either 0.9% NaCl (control injection) or recombinant adeno-associated viruses (rAAV) to overexpress the control protein luciferase (rAAV-Luc) or α-Syn (rAAV-α-Syn). Animals of each group were then randomized to be treated with once daily i.p. injections of either solvent or vinpocetine for a 9-week period. (**c**) Representative images of the substantia nigra, immunostained for tyrosine hydroxylase (TH, brown) to identify dopaminergic neurons in solvent-treated mice. Red ellipse: area of cell loss upon α-Syn overexpression. (**d**) Stereological quantification of the number of TH^+^ cells on the control (Ctrl) side and the stereotactic injection side of solvent treated mice. On the injection side, there was a significant loss of TH^+^ cells (red bar, right panel) after α-Syn overexpression. (**e**) Representative images of the TH^+^ substantia nigra of vinpocetine treated mice of the three injection groups. Red ellipse: in contrast to solvent treated mice, vinpocetine treated mice showed no loss of TH^+^ neurons upon α-Syn overexpression. (**f**) Quantification of the number of TH^+^ cells on the control side and the stereotactic injection side of vinpocetine treated mice. Vinpocetine completely prevented α-Syn-induced cell death. *P < 0.05, ***P < 0.001, n.s. not significant, two-way ANOVA with Tukey’s HSD post-hoc test. N-values, F-values, degrees of freedom (DF), and P-values: (**d**) *N* = 5 (NaCl groups), *N* = 7 (rAAV-Luc groups), *N* = 11 (rAAV-α-Syn groups), interaction: *F* = 10.79 DF = 40, *P* = 0.0002, i.p. treatment: *F* = 18.95, DF = 40, *P* < 0.0001, overexpression: *F* = 12.31, DF = 40, *P* < 0.0001. (**e**) *N* = 5 (NaCl groups), *N* = 6 (rAAV-Luc groups), *N* = 14 (rAAV-α-Syn groups), interaction: *F* = 0.16, DF = 44, *P* = 0.85, i.p. treatment: *F* = 0.32, DF = 44, *P* = 0.57, overexpression: *F* = 0.03, DF = 44, *P* = 0.97. Data are mean ± SEM. Scale bars: 1 mm.

We next aimed to study if vinpocetine would also protect against α-Syn-induced neuronal cell death *in vivo*. For this purpose, recombinant adeno-associated viral vectors (rAAV) were stereotactically injected unilaterally into the right substantia nigra of 11-week-old mice to overexpress human wild-type α-Syn (rAAV-α-Syn; Fig. 5a). rAAV expressing the control protein luciferase (rAAV-luc) or NaCl only were injected in other mice to control for procedural toxicity.

A subgroup of animals was sacrificed three weeks after injection to determine the transduction rate for rAAV-α-Syn and rAAV-luc (3 mice per group). The percentage of dopaminergic (i.e. tyrosine hydroxylase-immunoreactive (TH^+^)) neurons in the substantia nigra showing cytoplasmic human α-Syn immunoreactivity was 91.7 ± 0.6% after rAAV-α-Syn injection, but 0 in the control groups. The percentage showing luciferase immunoreactivity after rAAV-luc injection was 93.4 ± 1.6%, but 0 in the other groups. Thus, stereotactical injection of both rAAVs led to high transduction rates in the dopaminergic substantia nigra neurons of mice, with no significant difference between α-Syn and luciferase transduction.

The remaining mice of each group (rAAV-α-Syn, rAAV-luc, NaCl) were randomized to vinpocetine or solvent treatment (Fig. 5b). Treatment was conducted by once daily intraperitoneal (i.p.) injections of 25 mg/kg vinpocetine or the corresponding volume of solvent, starting one week after the rAAV injections for a period of 9 weeks (Fig. 5a). All animals survived the injection procedure and treatment period.

Ten weeks after stereotactical injection, the numbers of TH^+^ cells were determined in the substantia nigra and compared between the left side (no injection) and the right side (stereotactical injection). Nigral injection of NaCl in i.p. solvent-treated animals had no effect on the number of TH^+^ neurons (4108 ± 54 [left] vs. 4168 ± 92 [right], *P* = 0.99, Fig. 5c, upper panel and Fig. 5d, blank columns). Also rAAV-luc injection in i.p. solvent-treated mice did not affect the numbers of nigral TH^+^ neurons (3806 ± 182 [left] vs. 4063 ± 173 [right], *P* = 0.78, Fig. 5c, middle panel and Fig. 5d, yellow columns). However, α-Syn overexpression in i.p. solvent-treated mice, decreased the number of TH^+^ neurons in the substantia nigra by 28.1 ± 2.7% (4087 ± 130 [left] vs. 2915 ± 79 [right], *P* < 0.0001, Fig. 5c, lower panel and Fig. 5d, red columns). These data show that α-Syn overexpression, but not the overexpression of a control protein and not the stereotactical procedure *per se*, induced degeneration of TH^+^ neurons.

After i.p. vinpocetine treatment, there was expectedly no difference in the number of TH^+^ neurons on the side of the stereotactical injection in the NaCl and rAAV-luc injection groups (compare Fig. 5c, upper and middle panel, and Fig. 5e, upper and middle panel). Remarkably, there was also no significant loss of TH^+^ cells in the substantia nigra of rAAV-α-Syn injected mice treated with vinpocetine (Fig. 5e, lower panel, red circled area and Fig. 5f). Consistently, the number of TH^+^ cells in the rAAV-α-Syn-injected substantia nigra of vinpocetine-treated mice was significantly higher than the number of the rAAV-α-Syn-injected substantia nigra of solvent-treated mice (3870 ± 141 vs. 2915 ± 79, *P* < 0.001). Representative immunohistochemistry images with an α-Syn antibody in rAAV-α-Syn-injected mice, or with a luciferase antibody in rAAV-luc-injected mice are presented in Supplementary Fig. S4. Our findings indicate that systemic application of vinpocetine in mice largely prevented α-Syn-induced dopaminergic neuronal cell death in the substantia nigra *in vivo*.

Since whole brains were used for immunohistochemistry, we could not perform Western blots to quantify α-Syn overexpression levels. However, the toxicity levels observed upon overexpression of α-Syn suggests that overexpression levels were moderate. Moreover, the absence of toxicity upon luciferase shows that the effect was α-Syn-specific and not caused by the extensive overexpression of a foreign protein.

## Discussion

The absence of neuroprotective drugs for PD and other synucleinopathies is a big unmet medical need, because these diseases progressively impair the patients’ quality of life and functionality in activities of daily living. The identification of new therapeutic targets is therefore of great importance. Development of new drugs from scratch is a time- and cost-intense process. A resource-sparing alternative approach is the screening of drugs already approved for use in humans, to identify previously unrecognized neuroprotective effects, which might qualify them for being repositioned into new indications.

Since α-Syn-induced neurotoxicity appears to be the core pathophysiology of synucleinopathies, we performed a high-throughput screening of 1,600 FDA-approved drugs in cultured human postmitotic dopaminergic mesencephalic neurons using slowly progressive cell death, induced by moderate overexpression of wild-type α-Syn as read-out. We identified dipyridamole, an unspecific PDE inhibitor^25, 26^, to confer strong neuroprotection. However, neuroprotective efficacy of dipyridamole in human patients appears unlikely, since it does not penetrate the blood-brain barrier. A systematic analysis of specific inhibitors of PDE isoenzymes identified potent neuroprotective properties of the PDE1 inhibitor vinpocetine, which was not part of the screening library. siRNA-mediated silencing of PDE1A, and with inferior efficacy silencing of PDE1C, reproduced the protective effects of the pharmacological inhibitors, validating these as molecular targets. The downstream mechanisms of action appear to depend on an increase of cGMP, but not of cAMP, nor of sodium channels or L-type calcium channels, all of which are being modulated by vinpocetine^27–29^, since elevation of cGMP levels, but not specific modulators of the latter mechanisms reproduced the neuroprotective effect. L-type calcium channels contribute to mitochondrial oxidative stress by intermittent uncoupling of the mitochondrial membrane^32, 33^. Thus, isradipine is currently being tested as a disease-modifying therapy in PD (ClinicalTrials.gov: NCT02168842). However, our data suggest that these calcium channels did not contribute to the α-Syn induced toxicity observed in our cell model. Interestingly, vinpocetine reduced a 37 kDa oligomeric α-Syn band in a concentration-dependent manner and in correlation with the protective efficacy, drawing further attention to the potentially harmful nature of this α-Syn species^18^. Finally, vinpocetine also rescued dopaminergic midbrain neurons from α-Syn-induced cell death in mice *in vivo*. In conclusion, this work identifies that inhibition of PDE1A and/or PDE1C might be useful in the prevention or treatment of synucleinopathies.

Vinpocetine had previously been shown to protect from neurotoxicity induced by 1-methyl-4-phenyl-1,2,3,6-tetrahydropyridine (MPTP) and rotenone^34, 35^, used as models of PD. We show here for the first time that vinpocetine protects *in vitro* and *in vivo* from α-Syn-induced toxicity. Vinpocetine is a chemical derivative of vincamine, an extract from the lesser periwinkle plant. It is licensed as drug in many countries for disorders of the CNS, including cognitive impairment, epilepsy, and stroke. Hence, data regarding pharmacokinetics, pharmacodynamics, safety, tolerability, and side effects are available. Vinpocetine crosses the human blood-brain barrier and rapidly enters the brain^36^. Studies with ^11^C-radiolabeled vinpocetine showed that 3.7% of a systemically administered dose is measurable in the human brain^36^. In addition, brain areas of highest vinpocetine uptake comprise the thalamus, upper brainstem, occipital cortex and cerebellum^36^, which are highly relevant for synucleinopathies. Vinpocetine is generally well tolerated and has favourable safety data for the use in humans. In summary, vinpocetine is a promising drug to influence the course of synucleinopathies. However, more specific inhibitors of PDE1A and/or PDE1C might be preferable to increase efficacy and to reduce the risk of side effects.

PDE inhibition has been previously discussed as therapeutic approach for Huntington’s disease^37^ and Alzheimer’s disease^38^. It was also reported that inhibition of the cAMP-selective PDE7 protected dopaminergic neurons in a 6-hydroxydopamine model of PD *in vivo* ^39^. Moreover, PDE1 inhibition was discussed as an approach to improve neuronal plasticity and to be neuroprotective as treatment option for neurodegenerative diseases^40^. Our work demonstrates PDE1, particularly PDE1A and PDE1C to be specific molecular targets to prevent α-Syn-induced neurotoxicity. PDE1 isoforms in general can catalyse both, the degradation of cAMP and cGMP^41^. Unfortunately, we were unable to measure cAMP and cGMP levels in our model, because the high amounts of dibutyryl-cAMP in the culture medium render measurements of other cyclic nucleotides impossible. Our results with the nucleotide cyclase stimulators, however, suggest that increased cGMP, but not cAMP levels, were relevant for the protective effect. Consistently, previous studies showed that vinpocetine’s inhibitory efficacy is strongest on PDE1A, and that vinpocetine and PDE1A have high effects on cGMP levels, but only low or no effects on cAMP^42, 43^. In addition, dipyridamole has little effects on cAMP but mainly elevates cGMP levels^44^. Inhibition of the cGMP-selective PDE5 was not protective, since this was not expressed in our model and inhibition of PDE6 was not attempted, since there are no specific inhibitors available and expression levels were very low. Inhibition of the PDE9 was not protective in our model, but PDE9 appears to regulate the natriuretic-peptide coupled cGMP pool^45^. In contrast, PDE1 and the guanylate cyclase stimulator BAY41-2272 stimulate the NO-generated cGMP pool^46^. Thus, it is possible that only the NO-generated cGMP pool is relevant for the protective effect. In order to add more proof that PDE1 inhibition was the relevant mechanism of action, we treated cells with a combination of vinpocetine and the guanylate cyclase stimulator BAY41-2272. However, this treatment turned out to be toxic for α-Syn overexpressing cells (data not shown). Thus, we cannot rule out with absolute certainty that protection by vinpocetine could be mediated by another mechanism independent of PDE1 inhibition. Our data addressing the mechanisms of action (Fig. 4), however, strongly suggest that PDE1 inhibition is relevant to protect from α-Syn induced toxicity.

Remarkably, vinpocetine selectively reduced a 37 kDa oligomeric α-Syn species which was only present in α-Syn-overexpressing cells. This effect was very similar to the observation we previously made with the neuroprotective substance trifluoperazine^18^. It is possible that vinpocetine inhibited the formation of the 37 kDa α-Syn species from monomeric α-Syn, or reverts the 37 kDa α-Syn species to monomeric α-Syn, since the latter was increased by vinpocetine. These data might suggest that the 37 kDa α-Syn species is relevant for α-Syn induced toxicity in our model.

Since the band is sodium dodecyl sulphate (SDS) resistant, it is possible that it is part of a larger aggregate formed under pathological conditions. However, it is also possible that the band comprises a trimer of α-Syn. In line with that, in a previous study insoluble oligomers of a similar size (42 kDa) have been observed *in vivo* and were considered to be trimers^13^. A more recent study found an SDS resistant α-Syn band with a size of 36 kDa in the cytosolic fraction of PD brain extracts and in α-Syn overexpressing SH-SY5Y cells. In this study, a mass spectroscopy analysis confirmed the presence of α-Syn in this band. However, the exact nature (extended monomer, dimer, or others) was also not elucidated^47^. It is possible that these small oligomeric bands observed by others and ourselves are of the same nature and slight differences in size are caused by the usage of different protein ladders or gels. However, it is also possible that the 37 kDa band we observed may consist of α-Syn covalently bound to another protein. Thus, the investigation of the exact nature of this 37 kDa species is currently ongoing, but goes beyond the scope of the current study.

Furthermore, the molecular link between cGMP and the observed change in the α-Syn pattern will require further attention.

Prior high-throughput studies in α-Syn models have not identified the molecular target and mechanisms of action described in the current work. This difference may relate to the fact that we used human postmitotic dopaminergic mesencephalic neurons as model, as opposed to yeast or C. elegans in prior studies^48–52^. Also, we modelled mild and slow neurodegeneration by moderate overexpression of human wild type α-Syn, as opposed to tagged or mutant α-Syn in prior screening studies^48–52^. While the currently pursued approach using FDA-approved drugs tests comparably small numbers of interventions, it delivers drug candidates ready for use in humans, and usually pre-described hypothetical mechanisms of action. Larger scale siRNA or CRISPR/Cas libraries to interrogate the entire genome for innovative therapeutic targets, or to test drug-like compounds with high chemical diversity are promising to deliver further innovative modifiers of α-Syn-induced neurotoxicity.

In summary, we screened 1,600 FDA-approved drugs and identified an unspecific PDE inhibitor to protect from α-Syn toxicity. The ensuing investigations demonstrated that a specific PDE1 inhibitor protects dopaminergic neurons in α-Syn models *in vitro* and *in vivo*. Knock down experiments further specified that PDE1A, and less so PDE1C, are appealing targets to develop neuroprotective interventions against α-Syn-toxicity. This demonstrates that particularly inhibition of PDE1A is a promising target for the treatment of neurodegenerative synucleinopathies in human patients and encourages to develop more specific PDE1A inhibitors for this indication.

## Materials and Methods

### Experimental design

The aim of the study was to perform a high-throughput screening in an α-Syn cell model in order to identify compounds with the potential to protect from α-Syn induced toxicity. After the primary screening the top hit was further characterized regarding PDE inhibition as mode of action by using other more specific PDE inhibitors. Moreover, PDE1A inhibition was confirmed as mode of action, using siRNAs *in vitro*. Vinpocetine, a specific PDE1 inhibitor was chosen for validation in an α-Syn *in vivo* model.

### Low-throughput cell culture

LUHMES (Lund human mesencephalic) cells^53^ were cultured at 37 °C, 5% CO_2_, and 100% humidity, as described previously^18^. In brief, cell culture flasks (Nunclon Δ surface, NUNC A/S, Roskilde, Denmark) were pre-coated with 0.1 mg/ml poly-L-lysine (PLL; Sigma-Aldrich, St. Louis, MO, USA) over night at + 4 °C as substratum. PLL was removed by 3 times rinsing with phosphate-buffered saline (PBS). For proliferation LUHMES cells were plated in DMEM/F12 (Sigma-Aldrich) with 1% (v/v) N-2 supplement (Life Technologies, Carlsbad, CA, USA), and 0.04 µg/ml basic fibroblast growth factor (bFGF; R&D Systems, Minneapolis, MN, USA). For differentiation, 120,000 cells per well were transferred in 48-well plates (Nunclon Δ surface, NUNC A/S), pre-coated with 0.1 mg/ml PLL over night at +4 °C, rinsed three times with PBS, incubated with 5 µg/ml fibronectin (Sigma-Aldrich) over night at 37 °C, and rinsed once with PBS. Cells were differentiated in DMEM/F12 supplemented with 1% N-2 supplement, 2 ng/ml glial derived neurotrophic factor (GDNF, R&D Systems), 0.49 µg/ml N^6^,2′-O-dibutyryladenosine 3′,5′-cyclic monophosphate, and 1 µg/ml tetracycline. Forty-eight hours after plating, cells were transduced with adenoviral vectors (BioFocus DPI, Leiden, Netherlands), encoding human wild-type α-Syn or GFP as control protein under the CMV promotor, at a multiplicity of infections (MOI) of 2 relative to the number of seeded cells. Twenty-four hours thereafter, cells were rinsed three times with PBS to remove remaining virus particles.

### High-throughput cell culture

To achieve optimal homogeneity throughout the screening plates, LUHMES cells were plated in proliferation medium at 4,000,000 cells per 75 cm^2^ cell culture flasks, coated with PLL and fibronectin, as described above. After 24 hours, the medium was changed to differentiation medium and 24 hours thereafter, the cells were transduced with the adenoviral vectors encoding human wild-type α-Syn at a MOI of 5. The MOI was higher to adjust for proliferation in the first 24 hours after plating. Twenty-four hours after transduction, cells were rinsed three times with PBS to remove remaining virus particles. Cells were then detached by incubation with trypsin-EDTA solution (Sigma-Aldrich) for 5 min at 37 °C, and re-plated at 16,500 cells per well in 384-well multi-well plates.

### High-throughput screening

A library of 1,600 FDA-approved drugs (Pharmakon 1600, MicroSource Discovery Systems Inc., Gaylordsville, CT, USA) was screened at 3 µM and 10 µM each in triplicates. The compounds were delivered as 10 mM stock solutions, dissolved in dimethyl sulfoxide (DMSO). Stock solutions were aliquoted using the Biomek FXp laboratory automation station (Beckman Coulter, Brea, CA, USA) and dissolved in five times concentrations in medium with the Matrix Wellmate (Thermo Scientific, Waltham, MA, USA). Ten µl of these solutions were added to empty 384-well plates (NUNC A/S) using the Freedom EVO workstation (Tecan, Männedorf, Switzerland). Thereafter, 16,500 α-Syn transduced LUHMES cells in 40 µl medium were added in each well, using the Multidrop™ 384 Reagent Dispenser (Thermo Scientific). The final DMSO concentration never exceeded 0.1%. Transduced cells without further treatment or treated with DMSO alone were used as negative controls. To quantify cell death, 6 days after adenoviral transduction, 4 µg/ml propidium iodide (PI, Sigma-Aldrich) and 2 µg/ml Hoechst 33342 were added to the culture wells. After 15 min of incubation four images per well were taken using the Opera High Content Screening System (Perkin Elmer, Waltham, MA, USA). The percentage of living cells without PI incorporation was determined relative to all Hoechst 33342^+^ cells. In preliminary experiments, we identified that 1 mM caffeine was protective in our cell model. This condition was used as protective positive control in the initial runs of the screening. After identifying that dipyridamole was protective, we used this as positive control.

The modified Z-score of the survival rate (%) was determined in α-Syn transduced, DMSO- or compound-treated cells by computing the median and median absolute deviation. All wells with a Z-score > 2.5 were considered as positive hits. All wells with a Z-score < −3 were considered as negative hits. The false positive rate was determined by the percentage of DMSO-treated wells with a Z-score > 2.5. The false negative rate was determined by the percentage of DMSO-treated wells with a Z-score < −3.

### Gene expression analysis

RNA was extracted using β-mercaptoethanol (Sigma-Aldrich) activated RLT buffer (Qiagen, Hilden, Germany) from three replicates of cells grown according to the low-throughput protocol 4 days after transduction with adenoviral vectors to express α-Syn. The expression analysis was performed with Illumina HumanHT-12_V3 bead chips (Illumina, San Diego, CA, USA). Detection p-values were processed by the Genome Studio Software. Probes with values below 0.05 were considered as expressed in the respective sample.

### Treatment with PDE inhibitors, cation channel inhibitors, and nucleotide cyclase stimulators

Cells were plated, differentiated, and transduced according to the low-throughput protocol. After removal of adenoviral vectors, fresh differentiation medium was added containing different PDE inhibitors [PDE1: vinpocetine, PDE2: Bay 60-7550, PDE3: milrinone, PDE4: rolipram, PDE5: sildenafil, PDE7: BRL 50481, PDE8: PF-4957325-00, PDE9: Bay 73-6691], dissolved in DMSO. In the same way tetrodotoxin (Sigma-Aldrich), isradipine (Sigma-Aldrich), forskolin (Sigma-Aldrich), and BAY41-2272 (Sigma-Aldrich) were dissolved in DMSO. The final DMSO concentration never exceeded 0.2%. Control cells were treated with DMSO alone.

### Lipofection of siRNAs

Stealth siRNAs (Thermo Scientific) against PDE1A, PDE1B, PDE1C, and a control siRNA were incubated in OptiMem medium (Thermo Scientific) with lipofectamine RNAiMax (Thermo Scientific) for 20 min to form complexes. Cells, cultured and transduced according to the low-throughput protocol, were treated with lipofectamine-siRNA complex after removal of adenoviral vectors. The final concentration of the siRNAs and lipofectamine RNAiMax was 50 nM and 2 µl/ml, respectively.

### Quantitative Real-Time PCR

RNA from LUHMES cells grown in 6-well plates was extracted using the RNeasy Mini Kit (Qiagen, Hilden, Germany) according to the manufacturer’s instructions. RNA concentration was determined on a NanoDrop 2000 (Thermo Fisher) and equal amounts of RNA were transcribed to cDNA using the iScript^TM^ Reverse Transcription Supermix (Bio-Rad Laboratories, Berkeley, CA, USA) according to the manufacturer’s instructions. Forty cycles of Real-Time PCR from cDNA equivalent to 25 ng RNA/replicate were performed on the Applied Biosystems StepOnePlus system in triplicates using TaqMan Universal Master Mix II and TaqMan primers (Thermo Fisher) against *PDE1A* (Hs00897273_m1), *PDE1B* (Hs00354773_m1), and *PDE1C* (Hs01095682_m1). *PSMC1* (Hs02386942_g1) and *PPIA* (Hs04194521_s1) were used as reference genes for relative quantification. All quantities were computed relative to untreated LUHMES cells using the ∆∆CT method.

### Measurement of LDH release to determine cell death

In the low-throughput experiments, cell death was quantified by measurement of lactate dehydrogenase (LDH) released into the culture medium six days after transduction, using the CytotoxOne Membrane Integrity Assay (Promega, Fitchburg, WI, USA) according to the manufacturer’s instructions. Fluorescence was quantified with a Fluostar Omega plate reader (BMG Labtech, Ortenberg, Germany).

### Quantification of vital cells

Six days after transduction cells were fixed with 4% paraformaldehyde (PFA) for 15 min, then incubated with 1 µg/ml 4′,6-diamidino-2-phenylindole (DAPI) in PBS for 5 min, and washed three times with PBS. Five images per well from at least 12 wells per condition of at least 3 different biological replicates were taken in an automatized manner using an inverted microscope (DMI6000, Leica Microsystems) using Leica Application Suite AF. Counting of vital, non-apoptotic and non-pyknotic cells was performed using the cell counter plug-in of Fiji (Fiji Is Just ImageJ) 1.49 m for Windows 64-bit (http://fiji.sc/Fiji37) after blinding image data with Ant Renamer 2.10 (Antoine Potten, Brussels, Belgium).

### Immunocytochemistry

After fixation with 4% PFA cells were blocked and permeabilised in 5% horse serum with 0.3% Triton X-100 in PBS, and then were incubated with the primary antibody (rabbit anti-α-Syn [14H2L1, 701085, Life Technologies]; mouse anti β-III-tubulin [TU-20, EMD Millipore, Billerica, MA, USA]) for 2 hours at room temperature. Then the cells were washed three times with PBS, followed by incubation with a fluorescence labelled secondary antibody (anti rabbit Alexa 488-conjugated [Thermo Scientific], anti mouse Alexa 594-conjugated [Thermo Scientific]) for 1 hour, followed by DAPI staining as described above, and another three times washing. Images were taken using an inverted laser scanning confocal microscope (Zeiss LSM 880, Carl Zeiss, Oberkochen, Germany), using a 40x oil immersion objective and the ZEN Software (Carl Zeiss).

### Visualization of caspase 3 and 7 activity

Caspase 3/7 activity was visualized using CellEvent™ Caspase-3/7 Green Detection Reagent (Life Technologies). Therefore, the CellEvent™ stock solution (2 mM in DMSO) was added to the medium to achieve a final concentration 2 µM. After 30 min of incubation at 37 °C/5% CO_2_, cells were fixed with PFA, and incubated with DAPI as described above. Images were taken as described above.

### Western blot

Western blots were performed as previously described^18^. In brief, cell samples were lysed using the M-PER protein extraction buffer (Thermo Scientific Pierce Protein Research Products, Rockford, IL, USA). After extraction, the samples were centrifuged at 13,000 g for 15 min. Supernatants were loaded with Rotiload 1 loading buffer (Carl Roth, Karlsruhe, Germany) with 10 µg protein per lane on 12.5% sodium dodecyl sulphate (SDS) gels. Proteins were separated by gel electrophoresis and blotted onto nitrocellulose membranes. The membranes were blocked with 10% Rotiblock (10x, Carl Roth) in PBS with 0.1% polyethylene glycol sorbitan monolaurate (Tween® 20, Sigma-Aldrich) for at least 1 h at room temperature. Then, they were incubated with the respective primary antibody [rabbit anti-α-Syn (C-20, sc-7011-R, Santa Cruz Biotechnology, Santa Cruz, CA, USA; 1:1500); rabbit anti-α-Syn (14H2L1, 701085, Life Technologies; rabbit anti GFP (XP D5.1, 2956, Cell Signaling Technologies, Danvers, MA, USA; mouse anti-beta-actin (08691001, MP Biomedicals, Santa Ana, CA, USA; 1:10,000)], diluted in PBS with 10% Rotiblock and 0.1% polyethylene glycol sorbitan monolaurate. Then, membranes were washed three times for 10 min in PBS with 0.1% polyethylene glycol sorbitan monolaurate. Then they were incubated with horseradish peroxidase (HRP)-conjugated secondary antibodies [anti-mouse IgG (PI-2000, Vector Laboratories, Burlingame, CA, USA; 1:3000), anti-rabbit IgG (PI-1000; Vector Laboratories, 1:3000)]. Bound antibodies were visualized using Pierce ECL Western Blotting substrate (Thermo Scientific Pierce Protein Research Products, Rockford, IL, USA) on films (Carestream Kodak BioMax light film, Z373508, Sigma-Aldrich), the Chemidoc-XRS system (Bio-Rad Laboratories), or the ODYSSEY Fc instrument (LI-COR Biosciences, Lincoln, NE, USA). Films were digitalized using an Epson V33 flatbed scanner (Seiko Epson Corporation, Suwa, Japan). For quantification of Western blot band densities, the measurement tool in Fiji was used.

### Animal experiment

Fifty-eight male C57/BL/6 mice (Charles River Laboratories Germany, Sulzfeld, Germany), 10 weeks of age at the beginning of the experiment, were housed in standard cages at 23 °C with a 12:12 hours light/dark cycle, and *ad libitum* access to food and water. Animal experiments were performed according to German legislation and handled according to the EU Council Directive 86/609/EEC. All experimental procedures were approved by the appropriate governmental authority (Regierungspräsidium Gießen, Germany V54-19 c 20 15 h 01 MR 20/15 Nr. 1/2013).

### Unilateral α-Syn overexpression in mice

After one week of habituation, mice were deeply anesthetized by s.c. injection of ketamine (80 mg/kg; MSD Animal Health Intervet International, Unterschleißheim, Germany) and xylazine (4 mg/kg; Rompun^®^, Bayer, Leverkusen, Germany) in 0.9% NaCl and placed in a stereotaxic frame (David Kopf Instruments, Tujunga, CA, USA). Recombinant adeno-associated viral vectors (rAAV) expressing either human wild type α-Syn (rAAV-α-Syn, 1 × 10^13^ vg/ml in 0.9% NaCl) or luciferase (rAAV-luc, 1 × 10^13^ vg/ml in 0.9% NaCl) under control of the chicken beta actin (CBA) promoter were obtained from the Michael J. Fox Foundation. Two µl solution with rAAV-α-Syn or rAAV-luc (non-toxic control protein), or sterile saline (NaCl-controls) were infused within 10 min using a microsyringe with stainless needle (35 G, World Precision Instruments, Sarasota, FL, USA) and a micro pump (UltraMicroPump III with Micro4^TM^ digital controller, World Precision Instruments). The flow rate was 200 nl/min. Infusion was performed unilaterally into the right substantia nigra 3.1 mm posterior and 1.2 mm lateral from bregma, 4.2 mm ventral from the dura; in a flat skull position^54^. The needle stayed in the brain to facilitate diffusion for 5 minutes, before it was slowly retracted.

### Treatment of the mice

Mice of the three operation groups (rAAV-α-Syn, rAAV-luc, NaCl) were randomly assigned to be treated with daily i.p. injections of 25 mg/kg vinpocetine or solvent only. 1.5 mg vinpocetine (Tokyo Chemical Industry, Tokyo, Japan) was dissolved in 1 ml distilled water containing 2 mg/ml tartaric acid (Sigma-Aldrich) and 20 mg/ml 1,2-propylengylcol (Sigma-Aldrich). An appropriate volume was injected every day to reach a daily dose of 25 mg/kg vinpocetine. In the solvent groups the equivalent volume of solvent was injected. Group sizes were as follows: rAAV-α-Syn/solvent *n* = 11; rAAV-α-Syn/vinpocetine *n* = 14; rAAV-luc/solvent, *n* = 7; rAAV-luc/vinpocetine, *n* = 6; NaCl/solvent, *n* = 5; NaCl/vinpocetine, *n* = 5. Treatment started one week after surgery with a duration of 9 weeks. Then, the mice were sacrificed to determine the degree of neurodegeneration. Additionally, *n* = 3 rAAV-α-Syn and rAAV-luc mice were not treated, but sacrificed three weeks after virus injections to determine the transduction rate. Before sacrifice, mice were deeply anesthetized, as described above, and perfused transcardially with ice-cold 0.1 M PBS followed by 4% PFA in 0.1% phosphate buffer (PB).

### Immunohistochemistry

Coronal slices of the striatum and the SN were cut at 30 µm in 10 series on a cryostat microtome (Leica Microsystems) and stored at −20 °C in antifreeze buffer, containing 1:1:3 volume ratios of ethyl glycerol, glycerol and 0.1 M PB until further analysis. Slices were pre-incubated in 5% normal donkey serum (NDS) and 0.3% Triton X-100 in 0.1 M PB for 30 min. Then they were incubated over night at 4 **°**C in 0.1 M PB supplemented with 5% NDS, 0.3% Triton X-100, and the following primary antibodies: rabbit anti-TH, (1:1000, Thermo Scientific, Rockford, USA), rabbit anti-human α-synuclein (1:1000, EMD Millipore), goat anti-firefly luciferase (1:5000, Abcam, Cambridge, United Kingdom). Slices were then rinsed three times with 0.1 M PB followed by incubation with biotinylated species-specific secondary antibodies for 2 hours: donkey anti-rabbit/-goat, 1:1000 (Jackson ImmunoResearch Laboratories Inc., West Grove, PA, USA), and a one-hour incubation in avidin-biotin-peroxidase solution (ABC Elite, Vector laboratories, Burlingame, CA, USA). To visualize bound antibodies slices were incubated with 5% 3,3′-diaminobenzidine (DAB, Serva, Heidelberg, Germany) and 0.02% H_2_O_2_ in 0.1 M PB for 2 min, followed by three times rinsing with 0.1 M PB. Slices incubated as described above with the primary antibodies omitted were used to exclude unspecific binding. DAB stained slices were mounted on microscope slides, coated with mounting gel (Corbit-Balsam, Kiel, Germany), and covered with coverslips.

### Nissl staining

Free-floating slices of the SN were washed with 0.1 M PB and mounted on the gel-coated microscope slides. The slices were dried for at least three hours at room temperature and were dehydrated in a serial ascending concentration of ethanol (70%, 97%, 100%) and then rehydrated in a descending concentration of ethanol (100%, 97%, 70%). Thereafter, slices were incubated in 1% crystal violet for 2 min and dehydrated again as described above. Thereafter the slices were incubated in 5% xylene solution and covered with coverslips.

### Determination of the transduction rate of rAAV *in vivo*

Brain slices from animals sacrificed three weeks after intranigral injection of the respective rAAV vectors were double stained using antibodies against human α-Syn or anti-luciferase, respectively, followed by Nissl staining, as described above. In each of five slices of the SN anteroposterior at −2.86 mm, −3.01 mm, −3.16 mm, −3.31 mm, and −3.46 mm relative to the bregma, 200 random dopaminergic neurons were selected according to their morphology by Nissl staining. The transduction rate was quantified by the percentage of these cells showing immunoreactivity against human α-Syn or luciferase, respectively.

### Stereological determination of neurodegeneration

The number of TH^+^ neurons in the SN was counted using a stereology system (Microphot-FX, Nikon Corporation, Tokyo, Japan) with a 40x lens. Counting was performed with the slides encrypted and the person counting blinded to the experimental conditions. Cells were counted if the nucleus was present in the counting frame completely or touching the upper and/or right frame lines. Cells touching the lower and/or left frame line were not counted. The SN was outlined as on every 5th serial slice (2.4 to 4.1 mm dorsal of the bregma) and the total number of TH^+^ cells in the SN was determined with the Stereo Investigator software (MBF Bioscience, Williston, VT, USA) using the optical fractionator method.

### Statistical analysis

If not otherwise indicated, data were analysed using a one-way or two-way analysis of variance (ANOVA) test with Tukey’s HSD (Honestly Significant Difference) post-hoc test, as appropriate. The number of replicates was at least *n* = 3 for all experiments. All statistical analyses were performed with GraphPad Prism 6.0 (GraphPad Software, La Jolla, CA, USA) for Windows. *P* < 0.05 was considered statistically significant.

### Data availability

The data supporting the findings of this study are available from the corresponding authors on request.

## Electronic supplementary material

Supplemetary Information
