# Supplementary material for: Protective efficacy of phosphodiesterase-1 inhibition against alpha-synuclein toxicity revealed by compound screening in LUHMES cells
Source: Sci Rep. 2017 Sep 13;7:11469. doi: 10.1038/s41598-017-11664-5 (PMC5597612; doi:10.1038/s41598-017-11664-5)
Supplement: Supplementary file 1 — Supplemetary Information [file 41598_2017_11664_MOESM1_ESM.pdf]

Protective efficacy of phosphodiesterase-1 inhibition against alpha-synuclein toxicity revealed by compound screening in LUHMES cells

Matthias Höllerhage[1,2], Claudia Moebius[3], Johannes Melms[1,2], Wei-Hua Chiu[4], Joachim N. Goebel[4], Tasnim Chakroun[1,5] Thomas Koeglsperger[1,6], Wolfgang H. Oertel[4,7], Thomas W. Rösler[2,5,*], Marc Bickle[3,*], Günter U. Höglinger[1,2,5,*]

[1]Department of Translational Neurodegeneration, German Center for Neurodegenerative Diseases (DZNE), D-81377 Munich, Germany.

[2]Department of Neurology, Technical University of Munich, D-81675 Munich, Germany.

[3]HT-Technology Development Studio, Max Planck Institute of Molecular Cell Biology and Genetics, D-01307 Dresden, Germany.

[4]Department of Neurology, University of Marburg, D-35043 Marburg, Germany.

[5]Munich Cluster for Systems Neurology (SyNergy), D-81337 Munich, Germany

[6]Department of Neurology, Ludwig Maximilian University of Munich, D-81377 Munich, Germany.

[7]Institute of Neurogenomics, Helmholtz Center Munich, D-85764 Neuherberg, Germany.

[*]These authors codirected this work and contributed equally to this work.

Correspondence and request for materials should be addressed to G.U.H. (email: guenter.hoeglinger@dzne.de).

Supplementary Information

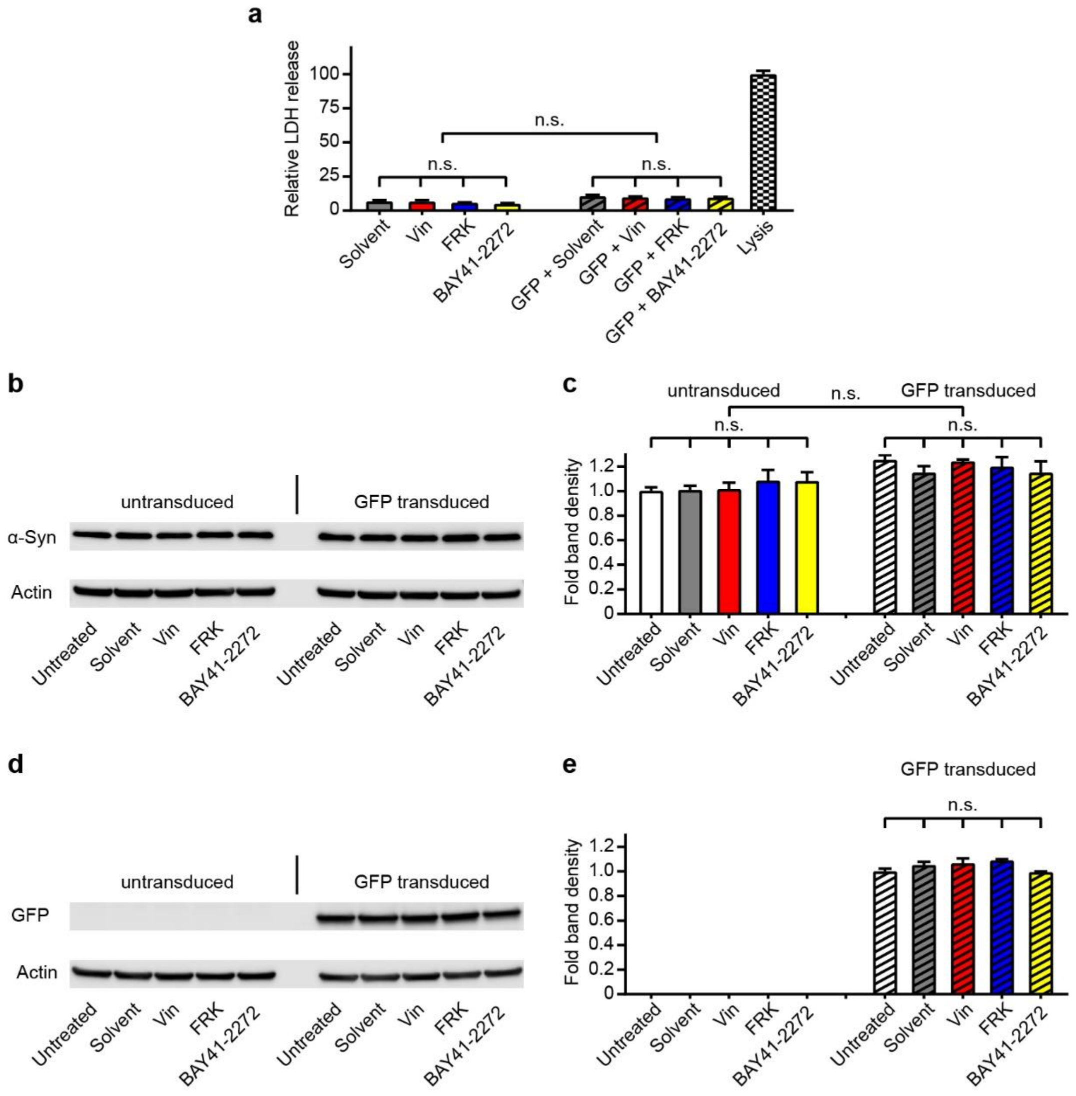

Supplementary Figure S1 Effect of vinpocetine, forskolin, and BAY41-2272 on control cells. (**a**) There was no difference in LDH release in untransduced cells treated with solvent (DMSO, grey bar), vinpocetine (Vin, 20 µM, red bar), forskolin (FRK, 10 µM, blue bar), and BAY41-2272 (1 µM, yellow bar) and in cells transduced with adenoviral vectors to express green fluorescent protein (GFP), treated with solvent (striped grey bar), Vin (striped red bar), FRK (striped blue bar), and BAY41-2272 (striped yellow bar). There was also no significant

difference in LDH release between untransduced and GFP transduced cells. Data shown are normalized to LDH levels measured in lysed cells (checkered bar). These data indicate that neither treatment nor the transduction procedure were toxic to the cells. (**b**) Western blot with an antibody against α-Syn in untransduced cells untreated cells left untreated or treated with solvent, Vin, FRK, or BAY41-2272. (**c**) The quantification of the Western blot showed no significant difference in the levels of endogenous α-Syn between the different treatment conditions in untransduced or GFP transduced cells. (**d**) Western blot with an antibody against GFP of cells transduced with adenoviral vectors to express GFP, which were either left untreated or treated with solvent, Vin, FRK, or BAY41-2272. (**e**) The quantification of the Western blot showed no difference in GFP levels between the different treatment conditions, showing that these did not influence the transduction procedure or the expression of the transduced protein. This indicates that the protective effect observed with Vin and BAY41-2272 in α-Syn-transduced cells could not be attributed to an influence on the transduction procedure or the expression of the transduced protein. n.s. not significant, one-way ANOVA with Tukey's HSD post-hoc test (a, b, f, j), or two-tailed *t*-test (d, h). N-values, F-values and degrees of freedom (DF): (a) $N \geq 4$, $F = 517.7$, DF = 39, (c) $N = 3$, $F = 35.69$, DF = 22, (e) $N = 3$, $F = 2.751$, DF = 10.

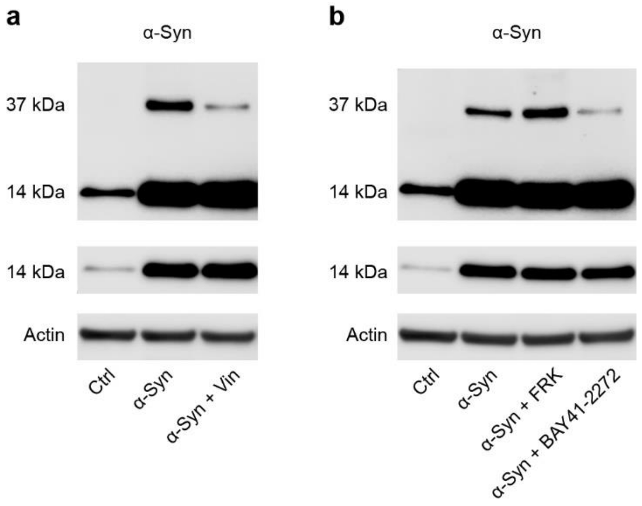

Supplementary Figure S2 Presence and reduction of a 37 kDa α-Syn band, demonstrated by a second anti- α-Syn antibody. (**a**) Representative Western blot with a second anti-α-Syn antibody (Life Technologies, 14H2L1) confirmed the presence of a 37 kDa α-Syn band after overexpression of human wild-type α-Syn using adenoviral vectors. Moreover, the treatment with vinpocetine (Vin) reduced this band. (**b**) Representative Western blot with a second α-Syn antibody (Invitrogen) confirmed that also treatment with BAY 41-2272, but not with forskolin (FRK), led to a reduction of the 37 kDa α-Syn band.

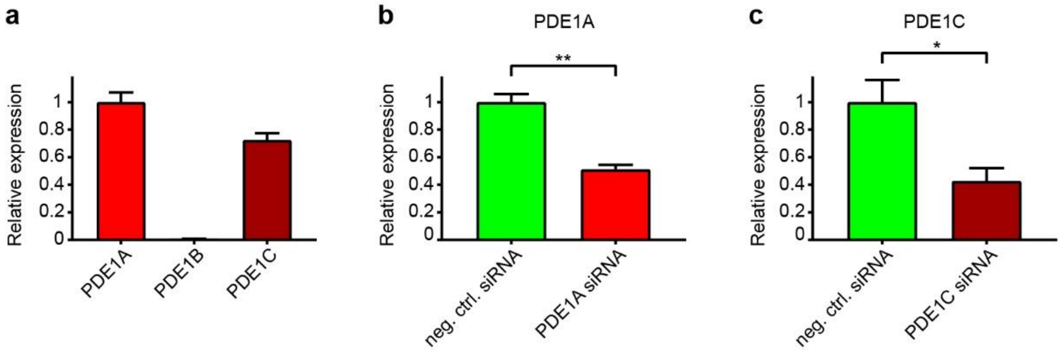

Supplementary Figure S3 Relative expression of PDE1 isoforms and efficacy of siRNA knockdown. (**a**) qPCR showed that PDE1A and PDE1C mRNA were present in LUHMES cells transduced with α-Syn expressing adenoviral vectors, while PDE1B mRNA levels were borderline to the detection threshold, confirming the Illumina chip analysis. Data were obtained four days after transduction. (**b**) qPCR showed that PDE1A mRNA levels were reduced by the siRNA against PDE1A compared to the negative control siRNA. (**c**) qPCR confirmed that PDE1C mRNA levels were reduced by the siRNA against PDE1C compared to the negative control siRNA. *$P < 0.05$, **$P < 0.01$, two-tailed *t*-test. N-values: (a) $N = 4$, (b) $N = 3$, (c) $N = 3$.

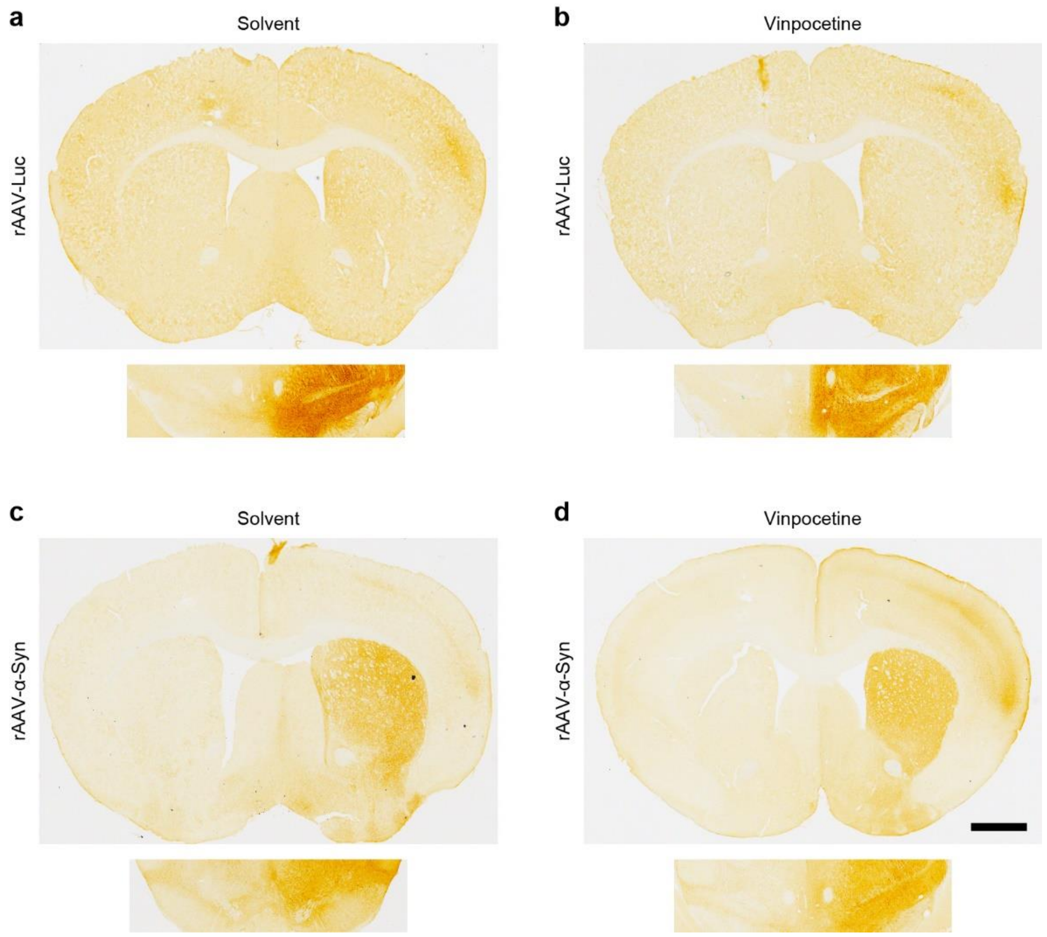

Supplementary Figure S4 Histological overviews of brain slices after injection of recombinant adeno-associated viruses into the substantia nigra. (**a**) The upper panels shows a staining with an anti-luciferase antibody of the striatum of mice 10 week after injection with rAAV-luc in the substantia nigra and treatment with solvent in the treatment period, the lower panel shows the substantia nigra. (**b**) The upper panel shows a staining with an anti-luciferase antibody of the striatum of mice 10 week after injection with rAAV-luc in the substantia nigra and treatment with vinpocetine in the treatment period, the lower panel shows the substantia nigra. (**c**) The upper panel shows a staining with an anti-α-Syn antibody of the striatum of mice 10 week after injection with rAAV-α-Syn in the substantia nigra and treatment with solvent in the treatment period, the lower panel shows the substantia nigra. (**d**) The upper panel shows a staining with an anti-α-Syn antibody of the striatum of mice 10 week

after injection with rAAV-α-Syn in the substantia nigra and treatment with vinpocetine in the treatment period, the lower panel shows the substantia nigra. Scale bar: 1 mm.

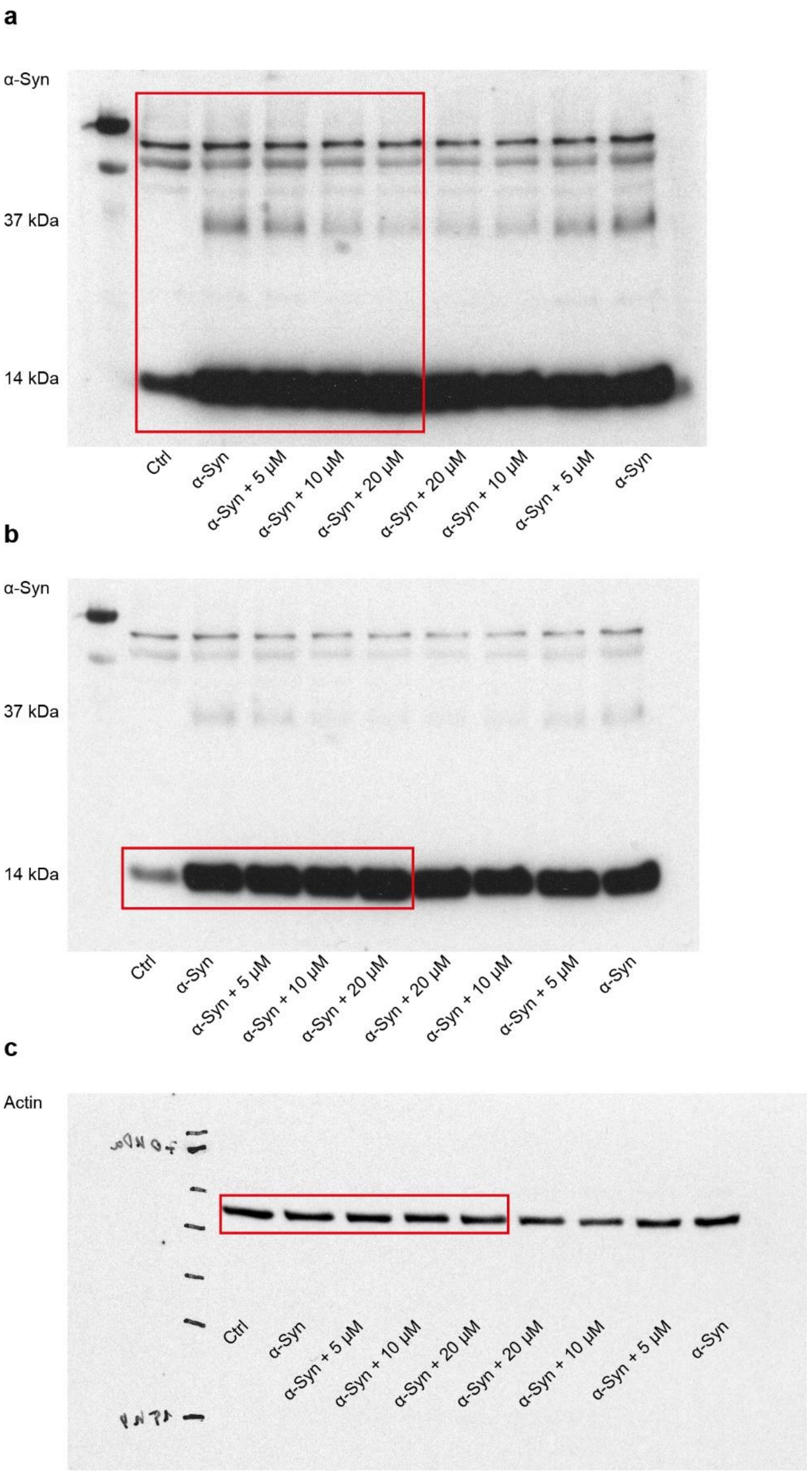

Supplementary Figure S5 Full-length Western blot from Figure 3 e. (**a**) High exposed image of the Western blot after staining with an antibody against α-Syn (C20, Santa Cruz). (**b**) Lower exposed image of the Western blot after staining with an antibody against α-Syn

(C20, Santa Cruz) (**c**). Image of the Western blot after staining with an antibody against beta-actin (08691001, MP Biomedicals).

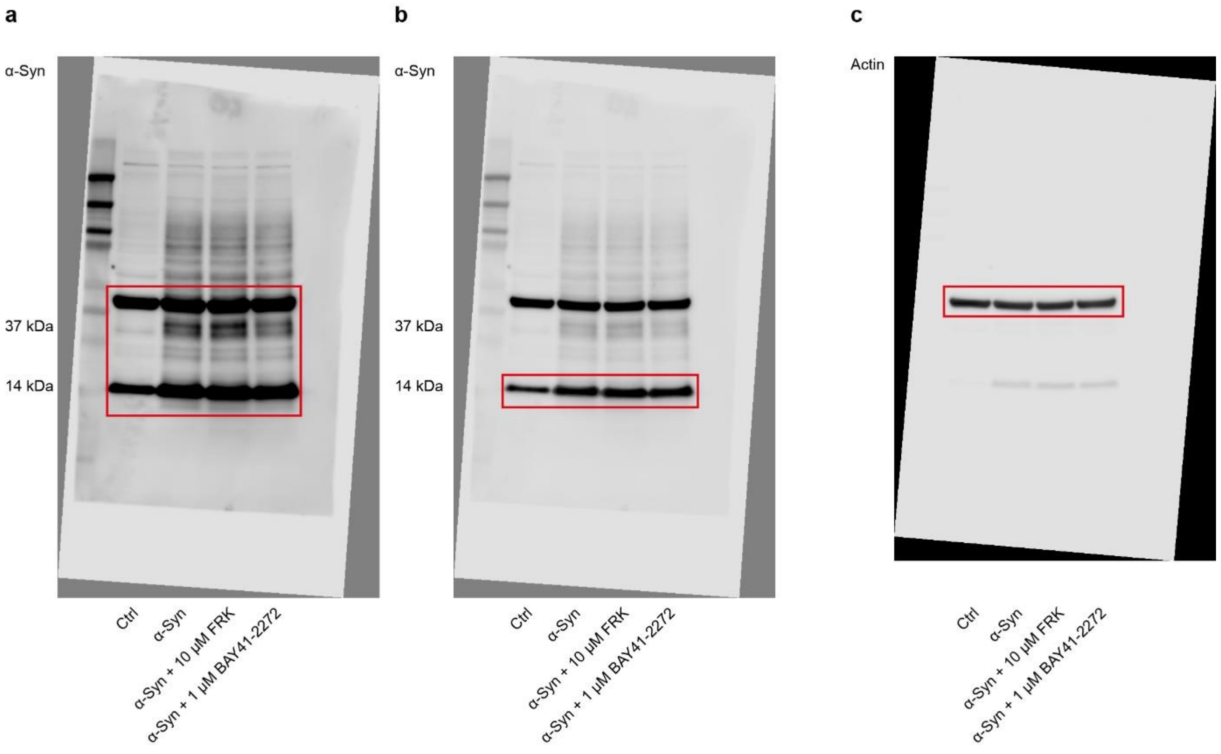

Supplementary Figure S6 Full length Western blot from Figure 4 d. (**a**) High exposed image of the Western blot after staining with an antibody against α-Syn (C20, Santa Cruz). (**b**) Lower exposed image of the Western blot after staining with an antibody against α-Syn (C20, Santa Cruz) (**c**). Image of the Western blot after staining with an antibody against beta-actin (08691001, MP Biomedicals).
